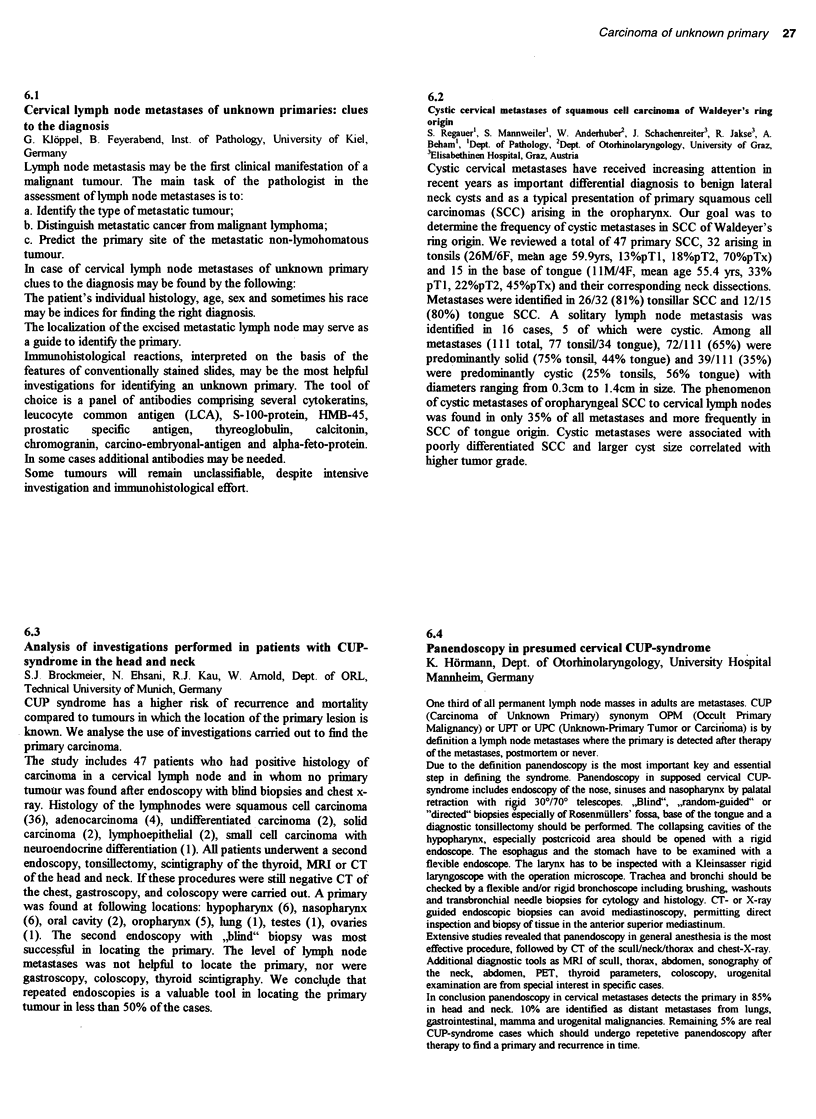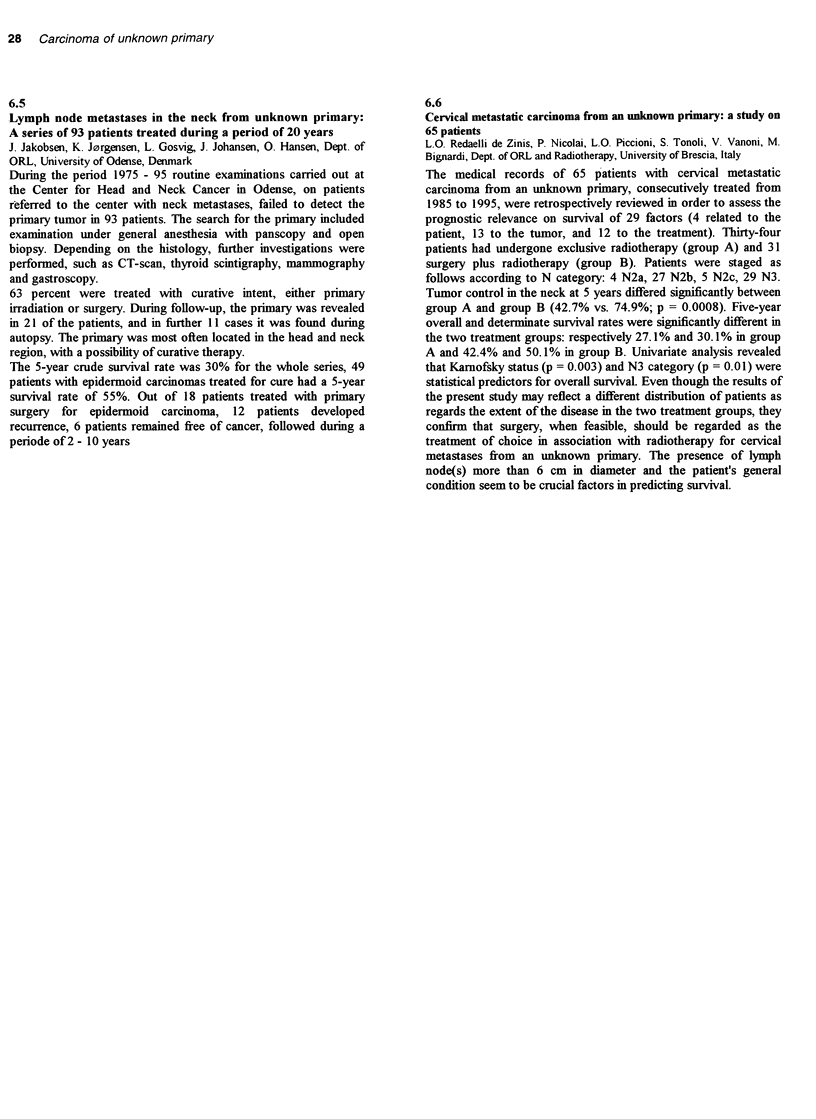# Carcinoma of Unknown Primary

**Published:** 1998

**Authors:** 


### 6.1
### Cervical lymph node metastases of unknown primaries: clues to the diagnosis

G. Klöppel, B. Feyerabend, Inst. of Pathology, University of Kiel, Germany

Lymph node metastasis may be the first clinical manifestation of a malignant tumour. The main task of the pathologist in the assessment of lymph node metastases is to:

a. Identify the type of metastatic tumour;
b. Distinguish metastatic cancer from malignant lymphoma;
c. Predict the primary site of the metastatic non-lymohomatous tumour.

In case of cervical lymph node metastases of unknown primary clues to the diagnosis may be found by the following:

The patient's individual histology, age, sex and sometimes his race may be indices for finding the right diagnosis.

The localization of the excised metastatic lymph node may serve as a guide to identify the primary.

Immunohistological reactions, interpreted on the basis of the features of conventionally stained slides, may be the most helpful investigations for identifying an unknown primary. The tool of choice is a panel of antibodies comprising several cytokeratins, leucocyte common antigen (LCA), S-100-protein, HMB-45, prostatic specific antigen, thyreoglobulin, calcitonin, chromogranin, carcino-embryonal-antigen and alpha-feto-protein. In some cases additional antibodies may be needed.

Some tumours will remain unclassifiable, despite intensive investigation and immunohistological effort.

### 6.2
### Cystic cervical metastases of squamous cell carcinoma of Waldeyer's ring origin

S. Regauer[1], S. Mannweiler[1], W. Anderhuber[2], J. Schachenreiter[3], R. Jakse[3], A. Beham[1], [1]Dept. of Pathology, [2]Dept. of Otorhinolaryngology, University of Graz, [3]Elisabethinen Hospital, Graz, Austria

Cystic cervical metastases have received increasing attention in recent years as important differential diagnosis to benign lateral neck cysts and as a typical presentation of primary squamous cell carcinomas (SCC) arising in the oropharynx. Our goal was to determine the frequency of cystic metastases in SCC of Waldeyer's ring origin. We reviewed a total of 47 primary SCC, 32 arising in tonsils (26M/6F, mean age 59.9yrs, 13%pT1, 18%pT2, 70%pTx) and 15 in the base of tongue (11M/4F, mean age 55.4 yrs, 33% pT1, 22%pT2, 45%pTx) and their corresponding neck dissections. Metastases were identified in 26/32 (81%) tonsillar SCC and 12/15 (80%) tongue SCC. A solitary lymph node metastasis was identified in 16 cases, 5 of which were cystic. Among all metastases (111 total, 77 tonsil/34 tongue), 72/111 (65%) were predominantly solid (75% tonsil, 44% tongue) and 39/111 (35%) were predominantly cystic (25% tonsils, 56% tongue) with diameters ranging from 0.3cm to 1.4cm in size. The phenomenon of cystic metastases of oropharyngeal SCC to cervical lymph nodes was found in only 35% of all metastases and more frequently in SCC of tongue origin. Cystic metastases were associated with poorly differentiated SCC and larger cyst size correlated with higher tumor grade.

### 6.3
### Analysis of investigations performed in patients with CUP-syndrome in the head and neck

S.J. Brockmeier, N. Ehsani, R.J. Kau, W. Arnold, Dept. of ORL, Technical University of Munich, Germany

CUP syndrome has a higher risk of recurrence and mortality compared to tumours in which the location of the primary lesion is known. We analyse the use of investigations carried out to find the primary carcinoma.

The study includes 47 patients who had positive histology of carcinoma in a cervical lymph node and in whom no primary tumour was found after endoscopy with blind biopsies and chest x-ray. Histology of the lymphnodes were squamous cell carcinoma (36), adenocarcinoma (4), undifferentiated carcinoma (2), solid carcinoma (2), lymphoepithelial (2), small cell carcinoma with neuroendocrine differentiation (1). All patients underwent a second endoscopy, tonsillectomy, scintigraphy of the thyroid, MRI or CT of the head and neck. If these procedures were still negative CT of the chest, gastroscopy, and coloscopy were carried out. A primary was found at following locations: hypopharynx (6), nasopharynx (6), oral cavity (2), oropharynx (5), lung (1), testes (1), ovaries (1). The second endoscopy with „blind" biopsy was most successful in locating the primary. The level of lymph node metastases was not helpful to locate the primary, nor were gastroscopy, coloscopy, thyroid scintigraphy. We conclude that repeated endoscopies is a valuable tool in locating the primary tumour in less than 50% of the cases.

### 6.4
### Panendoscopy in presumed cervical CUP-syndrome

K. Hörmann, Dept. of Otorhinolaryngology, University Hospital Mannheim, Germany

One third of all permanent lymph node masses in adults are metastases. CUP (Carcinoma of Unknown Primary) synonym OPM (Occult Primary Malignancy) or UPT or UPC (Unknown-Primary Tumor or Carcinoma) is by definition a lymph node metastases where the primary is detected after therapy of the metastases, postmortem or never.

Due to the definition panendoscopy is the most important key and essential step in defining the syndrome. Panendoscopy in supposed cervical CUP-syndrome includes endoscopy of the nose, sinuses and nasopharynx by palatal retraction with rigid 30°/70° telescopes. „Blind", „random-guided" or "directed" biopsies especially of Rosenmüllers' fossa, base of the tongue and a diagnostic tonsillectomy should be performed. The collapsing cavities of the hypopharynx, especially postcricoid area should be opened with a rigid endoscope. The esophagus and the stomach have to be examined with a flexible endoscope. The larynx has to be inspected with a Kleinsasser rigid laryngoscope with the operation microscope. Trachea and bronchi should be checked by a flexible and/or rigid bronchoscope including brushing, washouts and transbronchial needle biopsies for cytology and histology. CT- or X-ray guided endoscopic biopsies can avoid mediastinoscopy, permitting direct inspection and biopsy of tissue in the anterior superior mediastinum.

Extensive studies revealed that panendoscopy in general anesthesia is the most effective procedure, followed by CT of the scull/neck/thorax and chest-X-ray. Additional diagnostic tools as MRI of scull, thorax, abdomen, sonography of the neck, abdomen, PET, thyroid parameters, coloscopy, urogenital examination are from special interest in specific cases.

In conclusion panendoscopy in cervical metastases detects the primary in 85% in head and neck. 10% are identified as distant metastases from lungs, gastrointestinal, mamma and urogenital malignancies. Remaining 5% are real CUP-syndrome cases which should undergo repetetive panendoscopy after therapy to find a primary and recurrence in time.

### 6.5
**Lymph node metastases in the neck from unknown primary: A series of 93 patients treated during a period of 20 years**

J. Jakobsen, K. Jørgensen, L. Gosvig, J. Johansen, O. Hansen, Dept. of ORL, University of Odense, Denmark

During the period 1975 - 95 routine examinations carried out at the Center for Head and Neck Cancer in Odense, on patients referred to the center with neck metastases, failed to detect the primary tumor in 93 patients. The search for the primary included examination under general anesthesia with panscopy and open biopsy. Depending on the histology, further investigations were performed, such as CT-scan, thyroid scintigraphy, mammography and gastroscopy.
63 percent were treated with curative intent, either primary irradiation or surgery. During follow-up, the primary was revealed in 21 of the patients, and in further 11 cases it was found during autopsy. The primary was most often located in the head and neck region, with a possibility of curative therapy.
The 5-year crude survival rate was 30% for the whole series, 49 patients with epidermoid carcinomas treated for cure had a 5-year survival rate of 55%. Out of 18 patients treated with primary surgery for epidermoid carcinoma, 12 patients developed recurrence, 6 patients remained free of cancer, followed during a periode of 2 - 10 years

### 6.6
**Cervical metastatic carcinoma from an unknown primary: a study on 65 patients**

L.O. Redaelli de Zinis, P. Nicolai, L.O. Piccioni, S. Tonoli, V. Vanoni, M. Bignardi, Dept. of ORL and Radiotherapy, University of Brescia, Italy

The medical records of 65 patients with cervical metastatic carcinoma from an unknown primary, consecutively treated from 1985 to 1995, were retrospectively reviewed in order to assess the prognostic relevance on survival of 29 factors (4 related to the patient, 13 to the tumor, and 12 to the treatment). Thirty-four patients had undergone exclusive radiotherapy (group A) and 31 surgery plus radiotherapy (group B). Patients were staged as follows according to N category: 4 N2a, 27 N2b, 5 N2c, 29 N3. Tumor control in the neck at 5 years differed significantly between group A and group B (42.7% vs. 74.9%; $p = 0.0008$). Five-year overall and determinate survival rates were significantly different in the two treatment groups: respectively 27.1% and 30.1% in group A and 42.4% and 50.1% in group B. Univariate analysis revealed that Karnofsky status ($p = 0.003$) and N3 category ($p = 0.01$) were statistical predictors for overall survival. Even though the results of the present study may reflect a different distribution of patients as regards the extent of the disease in the two treatment groups, they confirm that surgery, when feasible, should be regarded as the treatment of choice in association with radiotherapy for cervical metastases from an unknown primary. The presence of lymph node(s) more than 6 cm in diameter and the patient's general condition seem to be crucial factors in predicting survival.